# Supplementary material for: Prognostic Relevance of Circulating Tumor Cells and Circulating Cell-Free DNA Association in Metastatic Non-Small Cell Lung Cancer Treated with Nivolumab
Source: J Clin Med. 2019 Jul 10;8(7):1011. doi: 10.3390/jcm8071011 (PMC6679117; doi:10.3390/jcm8071011)
Supplement: Supplementary file 1 [file jcm-08-01011-s001.pdf]

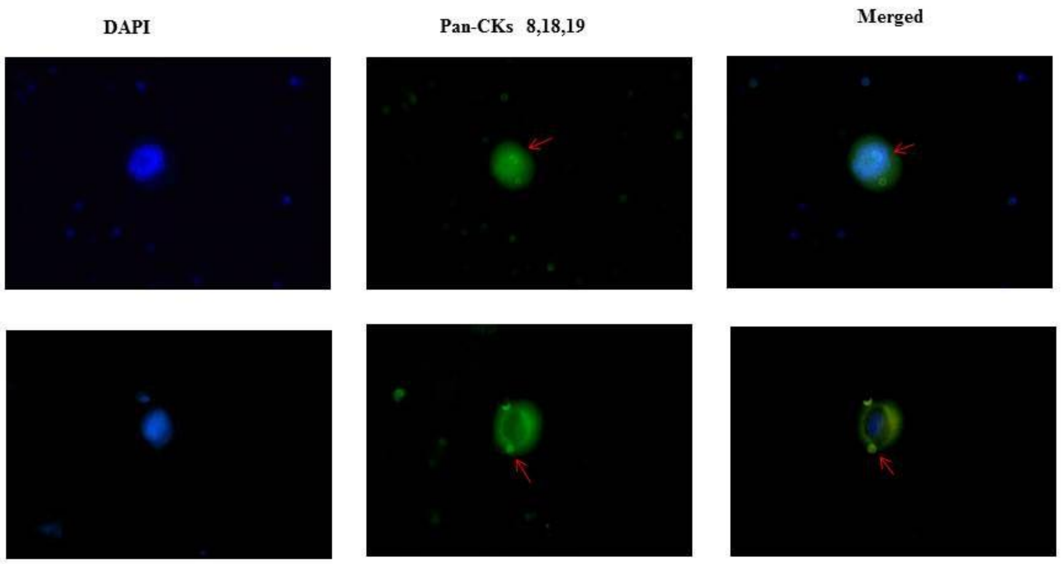

Figure S1. Immunofluorescence analysis of NSCLC cells entrapped on a ScreenCell Cyto device filter. Cells are stained with anti-CD45 , Pan-CKs 8,18,19 and 4′,6-diamino-2-phenylindole (DAPI). Cells with DAPI-positive nucleus, positive for CK staining in the cytoplasm, and negative for CD45 were considered to be CTCs. The red arrows indicate membrane pores (7.5 ± 0.36 μm).
